# Structural Variation (SV) Markers in the Basidiomycete *Volvariella volvacea* and Their Application in the Construction of a Genetic Map

**DOI:** 10.3390/ijms160716669

**Published:** 2015-07-22

**Authors:** Wei Wang, Bingzhi Chen, Lei Zhang, Junjie Yan, Yuanping Lu, Xiaoyin Zhang, Yuji Jiang, Taju Wu, Arend Frans van Peer, Shaojie Li, Baogui Xie

**Affiliations:** 1 Mycological Research Center, College of Life Sciences, Fujian Agriculture and Forestry University, Fuzhou 350002, China; E-Mails: uniwangwei@163.com (W.W.); cbz_2006@163.com (B.C.); zhanglei311540@163.com (L.Z.); junjie017@163.com (J.Y.); yuanplu1106@163.com (Y.L.); xyzhang8801@sina.com (X.Z.); arendvanpeer@gmail.com (A.F.P.); 2 College of Food Sciences, Fujian Agriculture and Forestry University, Fuzhou 350002, China; E-Mail: jyj1209@163.com; 3 State Key Laboratory of Mycology, Institute of Microbiology, Chinese Academy of Sciences, Beijing 100101, China; E-Mails: wutaju@163.com (T.W.); lisj@im.ac.cn (S.L.)

**Keywords:** SV marker, linkage map, *Volvariella volvacea*, genome re-sequencing

## Abstract

Molecular markers and genetic maps are useful tools in genetic studies. Novel molecular markers and their applications have been developed in recent years. With the recent advancements in sequencing technology, the genomic sequences of an increasingly great number of fungi have become available. A novel type of molecular marker was developed to construct the first reported linkage map of the edible and economically important basidiomycete *Volvariella volvacea* by using 104 structural variation (SV) markers that are based on the genomic sequences. Because of the special and simple life cycle in basidiomycete, SV markers can be effectively developed by genomic comparison and tested in single spore isolates (SSIs). This stable, convenient and rapidly developed marker may assist in the construction of genetic maps and facilitate genomic research for other species of fungi.

## 1. Introduction

DNA molecular marker systems are an important tool for studying genetic relatedness among individuals, population structure, phylogenetic relationships, constructing genetic maps and tracking quantitative traits (QTLs) [1]. Several DNA molecular marker systems were developed and applied. Restriction fragment length polymorphism (RFLP) was the earliest molecular marker [2]. However, the low sensitivity of this type marker results in a genetic map that contains large blanks. Random amplified polymorphic DNA (RAPD) is a fast and simple marker [3], but it is unstable and difficult to reproduce [4,5]. The sequence-characterized amplified regions (SCAR) marker is a dominant marker that was developed from RAPD but requires more work. It is more stable because it has a longer and more specific primer than RAPD [6]. The amplified fragment length polymorphism (AFLP) marker is simple and steady, but it requires high-quality genomic DNA [7]. Li and Quiros proposed a co-dominant sequence-related amplified polymorphism (SRAP) marker [8], which combines simplicity, reliability, a moderate throughput ratio and the facile sequencing of selected bands. The co-dominant trait is important for preventing false negatives. Even so, it can only amplify open reading frames (ORFs) and lost regions such as centromeres and telomeres. The simple sequence repeat polymorphism (SSR) marker is convenient and reliable, and it is used in gene mapping, cloning, and crop breeding [9,10]. However, the error rates associated with using markers that are based on simple sequence repeats are similar to RAPD markers [11]. The inter-sample sequence repeat (ISSR) marker that was developed from SSR is dominant [12]. Although they are generally thought to produce more repeatable results, ISSRs are less productive in terms of polymorphisms that are detected for some primer combinations [13]. With the development of genomic sequencing and the presence of abundant polymorphisms in genomes, single nucleotide polymorphism (SNP) markers [14,15] have been developed to create dense genetic linkage maps and genome-wide association studies [16].

A novel type of marker that is based on structural variation (SV) loci has been developed [17]. These markers, which are used in genome re-sequencing, are reportedly more specific to individuals than SNPs [18,19]. In normal, wild-type populations, approximately 5% of the genome is defined as SV and the size is equal to 250–300 genes [17]. The SV markers may represent the deletion, duplication, insertion, translocation or inversion of DNA segments in the genome and can profoundly affect the correlation between genetic and physical distance for the same intervals in plants [20]. Ren *et al.*, constructed a high-resolution genetic map of anchoring scaffolds in the sequenced watermelon genome [21]. Overall, 953 molecular markers, including 36 SV markers, were used in the linkage analysis, which suggested that SV markers could be applied to genetic map construction. Currently, there are no SV markers that have been developed in fungi.

With respect to the haploid fungi basidiomycete, which typically possesses small genomes and specialized life cycles, SV loci can easily be detected if two compatible strains’ genomes are sequenced and re-sequenced. A number of fruiting fungi have been sequenced or re-sequenced, such as *Laccaria bicolor* (*L. bicolor*) [22], *Schizophyllum commune* [23], *Coprinus cinereus* (*C. cinereus*) [24], *Ganoderma lucidum* [25,26], *Volvariella volvacea* (*V. volvacea*) [27,28] and *Flammulina velutipes* (*F. velutipes*) [29,30]. It would be useful to identify SV loci and develop SV markers by comparing these genomic sequences and re-sequencing.

A genetic linkage map is a useful tool in gene mapping, molecular breeding for genetic improvement, and genetic dissection of QTL [31,32]. In combination with analysis of the draft genome, these linkage maps can provide a scaffold for assembling a detailed physical map and can promote research in functional genomics [13,33]. *Volvariella volvacea*, also known as the Chinese straw mushroom, is an important edible fungus that is cultivated extensively across subtropical and tropical East and Southeast Asia [34]. Although *V. volvacea* has been cultivated for approximately 300 years and its genomic sequence is currently available [27,28], the number of chromosomes in the *V. volvacea* genome remains unconfirmed. Furthermore, no genetic map of *V. volvacea* has been constructed. The number of chromosomes still needs to be identified by genetic mapping or deep sequencing of *V. volvacea*.

In this study, we report a strategy to develop new type SV makers, and construct a genetic map of the basidiomycete *V. volvacea*. This application for SV markers from the genomic sequence may assist with *V. volvacea* genome assembly and genetic research. The construction method for genetic mapping in *V. volvacea* can also be applied to other basidiomycete species.

## 2. Results

### 2.1. Developed Structural Variation (SV) Markers

**Figure 1:**
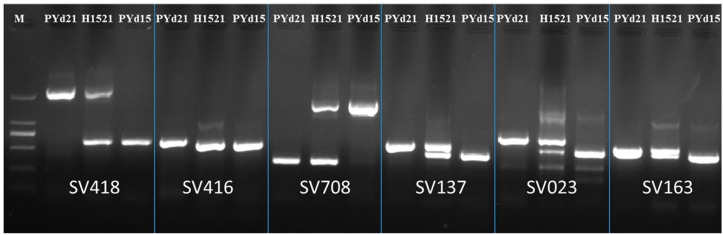
Examples of using SV markers in mapping polymorphisms in the selected strains of *V. volvacea*. M: DNA marker; DL2000.

After reads of re-sequenced genome (PYd15) were aligned to the reference genomic sequence (PYd21), a total of 35,389 SNPs, 1132 insertions-deletions and 943 SV loci were detected between the PYd21 and PYd15 strains. All of the SV loci were classified into four types: 559 Insertions, 371 Deletions, 11 Duplications, 1 Inversion and 1 Complex locus (Table 1 and Table S1). There were 182 scaffold distributions with SV loci within the 747,474 bp length (2% of genome) (Table 1 and Table S1). Based on these SV loci, 204 SV primer pairs were designed and tested. Examples of the use of SV markers are shown in Figure 1. Markers such as SV418, SV708, SV137, SV023 and SV163 were successfully amplified polymorphisms in these three strains. After eliminating non-polymorphic markers, a total of 104 SV markers were successfully applied to mapping the polymorphisms between the strains (Table S2 and Table S3).

**Table 1:** The statistics information of structural variation (SV) loci.

SV Kind	Number	Rate	Length
insertion	559	59.28	160,549
deletion	371	39.34	448,893
duplication	11	1.17	88,643
inversion	1	0.11	47,707
complex	1	0.11	1682
Total	943	–	747,474

### 2.2. Genetic Map Construction

A total of 235 viable single-spore strains were obtained from H1521 after fruiting. SCAR markers were used to distinguish homokaryotic from heterokaryotic strains, 192 homokaryons were identified and used as the mapping population.

**Figure 2:**
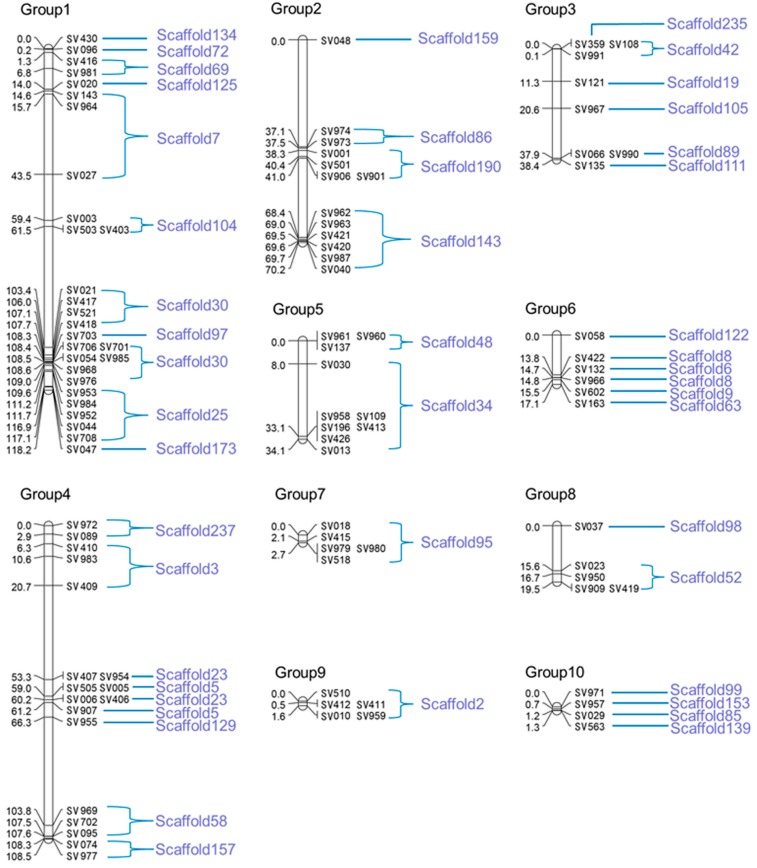
Genetic linkage map of *V. volvacea* constructed using SV markers.

The constructed genetic map consisted of 102 SV markers (two markers were not linked: SV011; SV978) that were distributed across ten linkage Groups, accounting for a total length of 411.61 cM. This is equivalent to a physical length of approximately 19.65 Mb or 52.8% of the genome (Total genome size: 37.2 Mb), with an average distance of 4.03 cM between adjacent markers (Figure 2). The number of markers on each linkage Group, the length of each linkage Group, and the average distance between adjacent markers on each linkage Group varied from 4 to 28, 1.295 to 118.211, and 0.32 to 6.03 cM, respectively (Figure 2, Table 2). The variation in the last parameter was not statistically significant.

**Table 2:** Summary of the obtained genetic linkage Groups.

Linkage Group	GD (cM)	No. of Markers	Average Marker Spacing (cM)	No. of Assembled Scaffolds	The Sizes of Assembled Scaffolds (kb)
Group 1	118.211	28	4.22	10	4543
Group 2	70.185	13	5.40	4	2742
Group 3	38.397	8	4.80	6	661
Group 4	108.488	18	6.03	7	3136
Group 5	34.148	10	3.41	2	2395
Group 6	17.118	6	2.85	5	391
Group 7	2.681	5	0.54	1	391
Group 8	19.473	5	3.89	3	3460
Group 9	1.605	5	0.32	1	880
Group 10	1.295	4	0.32	4	1048
SUM	411.601	102	4.04	43	19,647
AVERAGE	4.035	10	0.40	–	–

Altogether, 102 SV markers were localized to the linkage Groups. Nevertheless, our results showed the presence of variation in the frequency of genetic recombination (Figure 2). For example, the regions SV021–SV047 (14.8 cM) of linkage Group 1, SV962–SV040 (1.8 cM) of linkage Group 2, and SV422–SV163 (3.3 cM) of linkage Group 6 were areas of low recombination, whereas SV048–SV974 (37.1 cM) of linkage Group 2, and SV409–SV407 (32.6 cM) and SV955–SV969 (37.5 cM) of linkage Group 4 were zones with high recombination rates.

### 2.3. Scaffold Anchoring

The SV markers were developed using genomic sequence variations between PYd21 and PYd15. Thus, every SV marker could be positioned on the scaffold of the genomic sequences. Ten of the obtained linkage Groups contained 43 scaffolds (Table 2). Based on this result, we re-assembled the *V. volvacea* genome, and the number of scaffolds in the *V. volvacea* draft genome decreased from 302 to 269.

A total of 5172 genes were added to the linkage Groups after mapping the predicted genes from the genomic sequences. In the end, 44% of the genes (The total genes number = 11,534) [28] were located on the genetic map.

## 3. Discussion

Many studies have focused on converting RAPD markers into more stable and typically co-dominant markers such as SCARs or RFLPs to improve their reliability and reproducibility [2,35]. Kong developed 46 SCAR markers based on 155 polymorphic fragments from RAPD, SRAP and ISSR markers with a 29.7% success rate [36] in *V. volvacea*. In this study, we designed a total of 204 pairs of primers, 104 of which (51%) were successfully developed into molecular markers. As a novel type of marker, SV markers possess many advantages. First, SV markers can amplify unique polymorphic bands in a mapping population with linkage Groups. The amplified bands are easily detected using electrophoresis, which makes this a simple, time-saving and reproducible marker technique. Second, SV markers are very stable and convenient to use. They can be rapidly developed once the genome of the target organism is re-sequenced or has been sequenced multiple times. Third, the success rate of SV markers in constructing linkage Groups is higher than those of other markers. Because SV marker primers are designed from the flanking sequences of SV loci, amplified bands are always detectable in all single spores. SV loci and developed markers were used in constructing the high resolution genetic map of watermelon successfully [21]. However, the primers they designed located inside of SV loci was not co-dominance. In this study, primers located on the flanking sequences of SV loci are co-dominance and can prevent the occurrence of false negatives. Further, SV markers can be used in the construction of genetic maps, genetic diversity analyses and marking important traits, such as other molecular markers.

Although genetic maps for fungi were developed later than those of plants and animals, several linkage maps already exist for certain models or other important basidiomycete fungal species, including *Pleurotus ostreatus* [11], *C. cinereus* [37], *L. bicolor* [38], *Pleurotus pulmonarius* [39], *Agaricus bisporus* [40,41,42], *Pleurotus eryngii* [43], *F. velutipes* [44] and *Lentinula edodes* [45,46,47,48,49], using molecular markers other than SV markers. Several genetic maps of these basidiomycete fungi were combined with QTLs [41], elucidating karyotypes [44] and alignment to the whole-genome sequence assemblies [38]. Progress in the genetic characterization of *V. volvacea* remains poor, primarily due to a scarcity of information on its life cycle and the wide range of variation among its single-spore isolates [50,51,52,53]. We found two compatible monokaryotic strains demonstrating a heterothallic life cycle in *V. volvacea*. In this study, we constructed the first genetic map of *V. volvacea* using markers developed based on SV loci.

In combination with the first comprehensive genetic map of the *V. volvacea* genome and its sequence, SV markers will be useful in rapidly mapping genes that correspond to functions such as sexual processes and carbohydrate-active enzymes (CAZymes). We assigned the genomic sequence (scaffolds) to chromosomal regions based on SV markers that were identified in the genetic map, and the number of scaffolds in the *V. volvacea* draft genome decreased from 302 to 269. Therefore, it is also a beneficial method for genomic assembly. In other words, the genetic map used SV markers from genomic sequences and improved genomic sequences.

Certain scaffolds exhibited overlapping interspersed sequences. As shown in Figure 2, the SV703 marker in Group 1 was between the two adjacent markers SV706 and SV418 (Both belonging to scaffold 30), all of which were located on scaffold 97. Because the SV703 marker is in a densely labeled linkage Group and the genetic distance between the adjacent markers is short (approximately 0.7 cM), this phenomenon may be explained by the fact that recombination occurred at a lower frequency for these three loci in the mapping population and generated deviation. We can eliminate this phenomenon by enlarging the mapping population. Overall, a comparison of the sequences assembled from the obtained linkage Groups with those assembled using next-generation sequencing (NGS) revealed no significant differences. Recombination hotspots represent the chromosomal regions with higher recombination rates than the average rates in the genome [54]. If we combine the scaffolds with the linkage map, it is possible to identify recombination hotspots in the genome. For instance, SV143–SV027 (28.9 cM; ~242.6 kb) and SV410–SV409 (14.4 cM; ~152 kb) span a large scale on the linkage map and a relatively small region in the scaffold. In a similar way, recombination cold spots can also be identified, such as SV021–SV976 (5.6 cM; ~666 kb) and SV953–SV708 (7.5 cM; ~599 kb). Recombination hotspots with high recombination rates are of interest to researchers who focus on the times and traits of recombination.

The use of SV markers in molecular mapping may prove to be useful for mapping quantitative trait loci (QTLs) in *V. volvacea* in the future, particularly for mapping yield traits. This may be of significant value to the edible mushroom industry in Asia. Although relatively few markers (104) were identified among all of the linkage Groups, these would be sufficient, even in a large mapping population. As indicated in Table 2 and Figure 2, comparisons between the genetic and physical maps revealed that 1 cM is approximately equivalent to 47.7 kb, on average, in the *V. volvacea* genome. In future research, SV markers can be used in combination with other markers to successfully identify the genes and explain certain important traits. However, this is a new application of SV marker system in the sequenced basidiomycete *V. volvacea*.

The genome of fungi is not as large as those of plants and animals. Therefore, sequencing and re-sequencing the diminutive genome of fungi genome need less cost. SV markers could be easily developed based on genomic sequence. In basidiomycete, after mating two compatible homokaryotic strains that come from single spore isolates or protoplast monokaryogenesis, we can obtain SSIs from the fruiting body of the heterozygous heterokaryon. Both bipolar or tetrapolar basidiomycetes have a unique life cycle [55,56]. One homokaryotic strain should be sequenced and assembled, and the other compatible homokaryotic strain should be re-sequenced using the pair-end method. Then, we can detect the SV loci after mapping the re-sequenced reads to the referenced genome. The basidiospore in most basidiomycete species is haploid and can be treated as a mapping population. Genetic recombination can be detected using SV markers in the basidiospores. This successful method for genetic map construction in *V. volvacea* can also be applied to other fungi, at least in basidiomycete, and could benefit genetic mapping and genome research.

## 4. Experimental Section

### 4.1. Strains and Growth Conditions

**Figure 3:**
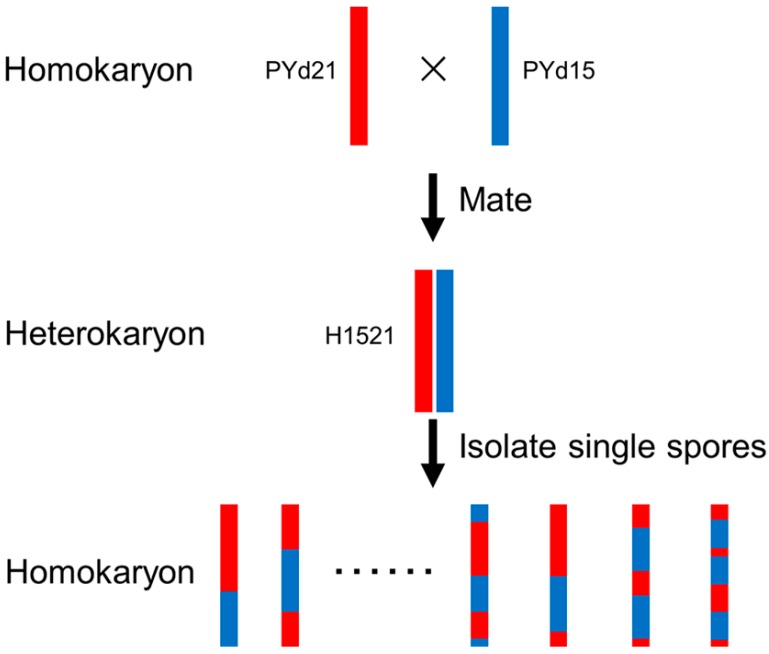
Construction of the single spore isolates (SSIs) mapping population in *V. volvacea*.

The *V. volvacea* strain PY1 is primarily cultivated in the province of Fujian in China and was used for initiating the experiment. The *V. volvacea* heterokaryotic strain H1521 was mated with the homokaryotic strains PYd15 and PYd21, both of which were isolated from the basidiospores of PY1. The mycelia of strain PYd21 do not produce any aerial hyphae, while PYd15 displays only a few. In contrast, H1521 produces large numbers of aerial hyphae. After H1521 fruited, single-spore strains were isolated to generate the mapping population. Next, we employed three SCAR markers (Table S4), using PCR to distinguish homokaryotic and heterokaryotic strains. Then, homokaryotic strains were used as a mapping population (Figure 3).

All strains were deposited in the Agricultural Culture Collection of China (PYd15: ACCC52631; PYd21: ACCC52632; H1521: ACCC52633), and maintained with periodic transfers on potato dextrose agar (PDA), at 20 °C.

The draft genome of *V. volvacea* PYd21 is available under accession no. PRJNA171553 at NCBI.

### 4.2. Genomic DNA Extraction and Genomic Sequencing

Genomic DNA of all of the aforementioned *V. volvacea* strains was isolated using a modified cetyl trimethylammonium bromide (CTAB) method [24]. The genomes of PYd21 and PYd15 were sequenced using a whole-genome shotgun strategy [28]. The strain PYd21 was sequenced *de* *novo* and assembled using the SOAP *de novo* [57] assembler (http://soap.genomics.org.cn/) while strain PYd15 was re-sequenced (Library of DNA fragments with insert sizes of 505 bp) [28]. Sample preparation and analytical processing (e.g., base calling) were performed by BGI-Shenzhen (http://www.genomics.cn).

### 4.3. Search SV Loci and Markers Development

To obtain SV loci, the two genomes were compared. All of the reads produced from the PYd15 library were aligned to the reference genomic sequence (PYd21), after which the coverage and depth distribution of re-sequencing were determined. Based on this, the SV loci between the homokaryotic strains were identified. For developing SV markers, we selected SV loci (Length: 200–800 bp) without base gaps (unconfirmed sequences) in the genomic sequence of PYd21. Based on the paired-end sequence method, one read could map to the plus strand, and the other read could map to the minus strand. The distance between two locations should be similar to the insert size. Therefore, the paired reads should have the correct direction and distance. SV loci can be detected from the abnormal reads. Each SV loci should be supported by abnormal reads from more than 5 pairs.

**Figure 4:**
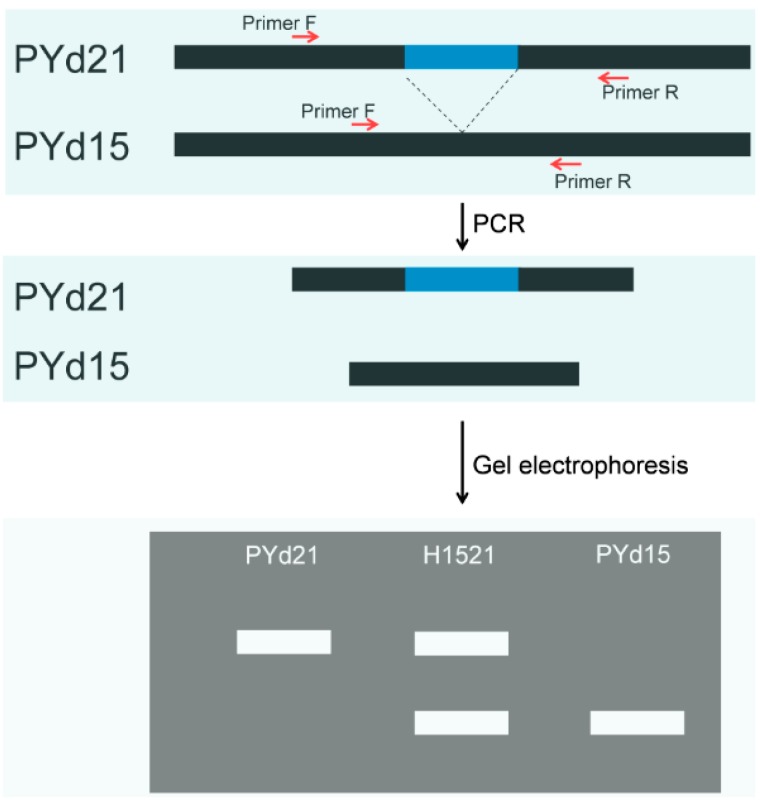
Schematic maps of SV loci and their PCR fragments. Primers used in PYd15 and PYd21 are same. Heterokaryon H1521 containing two types of nuclear (PYd15, PYd21) can generate two bands.

Primers were designed using Primer Premier 5 [58] based on the region including 300 bp flanking sequences on both sides of the SV loci. If a marker based on the heterokaryotic strain H1521 was able to amplify unique fragments from the homokaryotic strains PYd21 and PYd15, it was considered to be an effective marker (Figure 4).

### 4.4. PCR Amplification and Marker Scoring

All PCR amplification reactions were carried out in a 30 µL reaction mixture that contained 0.15 µg template DNA, PCR buffer (TaKaRa, Dalian, China), 3.9 mM of each dNTP, 10 µM primer, and 1.5 U Taq polymerase. The thermal cycling parameters were as follows: initial denaturation at 94 °C for 5 min, followed by 35 cycles of denaturation at 94 °C for 30 s, annealing at 60 °C for 30 s, extension at 72 °C for 40 s, and a 5 min final extension at 72 °C.

Markers that present in both of PYd15 and PYd21 (different size) and also present in the H1521 (both bands) were used for genetic mapping using the mapping population.

### 4.5. Linkage Analysis and Mapping

Effective SV markers were used in mapping population. A band’s size that matched PYd21 was marked “a” and one that was the same as PYd15 was marked “b” If the band was unclear, we marked it as “-”. After doing the statistics, linkage analysis was performed using the program MAPMAKER/EXP 3.0 [59,60] and incorporated Kosambi’s mapping function. All markers were assigned to chromosomes using the ASSIGN command with a LOD threshold of 3.0. For each chromosome, those markers with known positions in the physical map (version 5) were selected to construct a framework with the same marker order as that in the physical map. Subsequently, the TRY command was used to determine the locations of remaining markers in the framework. The distances between adjacent markers were calculated using the MAP command with error detection activated. The final genetic map was constructed using the program Mapdraw v. 2.1 [61].

Scaffold placements were determined based on the genetic map constructed in this study and consisted of 102 SV markers. The genome was re-assembled depending on the scaffold location on the genetic map.

## Supplementary Materials

Supplementary materials can be found at http://www.mdpi.com/1422-0067/16/07/16669/s1.
