# Supplementary material for: Structural Variation (SV) Markers in the Basidiomycete Volvariella volvacea and Their Application in the Construction of a Genetic Map
Source: Int J Mol Sci. 2015 Jul 22;16(7):16669–82. doi: 10.3390/ijms160716669 (PMC4519972; doi:10.3390/ijms160716669)
Supplement: Supplementary file 1 [file ijms-16-16669-s001.zip › ijms-91123-Supplementary Information/ijms-91123-Supplementary Table S1,S2,S4.pdf]

Supplementary Information

Table S1. SV loci numbers in each scaffold.

Scaffolds ID	SV Number
Scaffold1	2
Scaffold100	3
Scaffold101	5
Scaffold104	16
Scaffold106	3
Scaffold108	1
Scaffold11	2
Scaffold110	9
Scaffold111	3
Scaffold112	2
Scaffold113	3
Scaffold114	3
Scaffold115	8
Scaffold117	1
Scaffold118	5
Scaffold12	3
Scaffold121	2
Scaffold122	3
Scaffold123	1
Scaffold124	14
Scaffold125	4
Scaffold126	4
Scaffold127	3
Scaffold128	1
Scaffold129	8
Scaffold13	10
Scaffold130	4
Scaffold133	51
Scaffold134	3
Scaffold135	3
Scaffold138	2
Scaffold139	2
Scaffold14	3
Scaffold140	15
Scaffold144	1
Scaffold146	1
Scaffold147	1
Scaffold149	1
Scaffold15	2
Scaffold150	3
Scaffold151	1
Scaffold152	1
Scaffold153	1

Table S1. *Cont.*

Scaffolds ID	SV Number
Scaffold155	5
Scaffold157	4
Scaffold159	11
Scaffold16	2
Scaffold160	1
Scaffold161	1
Scaffold164	3
Scaffold166	1
Scaffold168	1
Scaffold169	8
Scaffold171	7
Scaffold172	4
Scaffold173	6
Scaffold175	3
Scaffold176	1
Scaffold177	4
Scaffold178	1
Scaffold179	1
Scaffold18	7
Scaffold180	2
Scaffold181	3
Scaffold182	2
Scaffold183	3
Scaffold185	2
Scaffold187	38
Scaffold189	7
Scaffold19	7
Scaffold190	19
Scaffold192	1
Scaffold193	1
Scaffold194	1
Scaffold198	2
Scaffold2	19
Scaffold20	11
Scaffold200	2
Scaffold201	8
Scaffold202	1
Scaffold203	1
Scaffold205	2
Scaffold209	1
Scaffold211	11
Scaffold212	6
Scaffold218	1
Scaffold22	2
Scaffold220	1

Table S1. *Cont.*

Scaffolds ID	SV Number
Scaffold225	4
Scaffold226	1
Scaffold229	1
Scaffold23	10
Scaffold230	3
Scaffold232	2
Scaffold235	2
Scaffold237	7
Scaffold239	9
Scaffold24	22
Scaffold244	1
Scaffold245	1
Scaffold248	1
Scaffold25	17
Scaffold26	8
Scaffold260	1
Scaffold263	1
Scaffold27	5
Scaffold271	3
Scaffold272	5
Scaffold275	3
Scaffold276	1
Scaffold277	1
Scaffold279	3
Scaffold28	3
Scaffold280	1
Scaffold283	1
Scaffold284	2
Scaffold29	3
Scaffold292	2
Scaffold3	14
Scaffold30	21
Scaffold31	3
Scaffold33	6
Scaffold34	21
Scaffold36	4
Scaffold37	3
Scaffold38	2
Scaffold4	5
Scaffold40	5
Scaffold41	4
Scaffold42	3
Scaffold44	4
Scaffold45	1
Scaffold46	4

Table S1. *Cont.*

Scaffolds ID	SV Number
Scaffold47	5
Scaffold49	2
Scaffold5	12
Scaffold50	5
Scaffold52	11
Scaffold53	6
Scaffold54	3
Scaffold55	6
Scaffold56	3
Scaffold57	3
Scaffold59	7
Scaffold6	3
Scaffold61	4
Scaffold64	10
Scaffold65	14
Scaffold66	9
Scaffold67	1
Scaffold68	2
Scaffold69	11
Scaffold7	12
Scaffold70	2
Scaffold71	4
Scaffold72	6
Scaffold73	8
Scaffold74	1
Scaffold75	3
Scaffold77	2
Scaffold78	5
Scaffold79	2
Scaffold8	6
Scaffold80	1
Scaffold81	12
Scaffold82	1
Scaffold83	3
Scaffold85	5
Scaffold86	5
Scaffold87	3
Scaffold89	16
Scaffold9	5
Scaffold90	2
Scaffold91	4
Scaffold92	2
Scaffold93	7
Scaffold94	6
Scaffold95	15

Table S1. *Cont.*

Scaffolds ID	SV Number
Scaffold96	3
Scaffold97	11
Scaffold98	5
Scaffold99	6

Table S2. The primer sequences of 104 SV markers.

Marker	Primer Sequences (5′–3′)
SV001F	GCGTTTAGTCTAGTGGATACT
SV001R	CCAAGCTCAGGATACACCAT
SV003F	AGAATGACGACTGCTGACGT
SV003R	ACGGAATGGTAGATGAGGCT
SV005F	AGGTGCTTGAACTCCAGCCT
SV005R	AGTGGCATGCTACATGTCCT
SV006F	GGAGAGTCTCAGAAGTGACT
SV006R	GTAGTAGGTCCCAGAATACTC
SV010F	ATGAGATGAGCGTCTGACGT
SV010R	TCGTCGTAGTCAACATCGTC
SV011F	TGCTACATGCCATCAATCCAG
SV011R	GAGCCTGACCGTGCTTACAG
SV013F	GTCTGTTGCCTTGACACCAT
SV013R	CTACAAGACTTGGCATCACCT
SV018F	CATGGATGTTCGTCTTCAAG
SV018R	ACGTTATCATGCCCGGATCT
SV020F	CATGGCCATAATCTGGAGCAT
SV020R	GTGCGGATGTGGCATTCTAT
SV021F	TGAGGATAGGATGTCAGCAG
SV021R	GGTCAATGACGATCAACTCCT
SV023F	ACAGTGTCGATGATGGTCAG
SV023R	GAGCTGGTATACACTACGGT
SV027F	GCATGACCACAGGACGATAT
SV027R	GTGATCAGTGGTTCGGTTAG
SV029F	TTGTCGAAGAGCCTGGCAGT
SV029R	CAGTTGTTGTATGCTGGAATG
SV030F	ACGTTGCTAGCTCATTTCCTT
SV030R	CACAACATCACAAGTAAGCTC
SV037F	GTAGCGTCTGAGATGTCTATG
SV037R	CAGGTTTCAGTTGTCATCGAG
SV040F	CAATCACTCTTCCGAGGCTC
SV040R	CCACTTTAACATGCGTATCC
SV044F	ACTCGATACACCGACCTAAG
SV044R	TGTAACACTCATCATGGAGCT
SV047F	ATGCCGAGGTCGAGGTTGTT
SV047R	ACGCGAGTGCGTGTGGCTT

Table S2. *Cont.*

Marker	Primer Sequences (5′–3′)
SV048F	ACTGACGAGCTTCTGCCCAT
SV048R	TTCATGTCTTCGGATGGAACT
SV054F	AGGCCTATGGCTTAGATTCT
SV054R	GGCCTAATGGTAATTGCTATG
SV058F	GCTCTTATCTTGATCTCCAG
SV058R	TGTGCTGAGTTCTCCTACAG
SV066F	ACGAACTCTCACCGCTACAC
SV066R	TCGAACCTCTTATCGCTGAC
SV074F	CCGGATGAATTCTTGTCGC
SV074R	ATCCAGGGATAAGCGTGCAT
SV089F	ACTGGACTGGGTAGCATTAC
SV089R	CGATTGGAACTGCGCTGCT
SV095F	GCCAGGGTATGAATGCCAAG
SV095R	GCAACAGCATCCGACTTGAC
SV096F	GTCAAGCAAGAACTGGTGCT
SV096R	GCTGTCCAGCTATCACTGT
SV108F	GCGATCGCAAAGAATCATTC
SV108R	AACATGATACGACCCACAGT
SV109F	GTATCACTGTCGGTATCCGT
SV109R	CTCCTGATACTGAGGTTCCT
SV121F	GACACTGTCAGTGGTTGCTC
SV121R	CAGCAAGATTGAGATGTGTG
SV132F	AGCTGCTCAAGAGTGTTAGC
SV132R	TGGCACTGTCTGCTTGGTTC
SV135F	GATGATGACCTTCTTGGTTC
SV135R	AACAACTTATGGTTGGTCCTC
SV137F	CGAGTCGCATTAACGCTGGT
SV137R	CGTCCTTGAGGACAACAGT
SV143F	AGCTTACAGGTATATGAGCGT
SV143R	CGAACATCCGCTTCAGCAAG
SV163F	AGCGTGAACGATGAATGAACT
SV163R	CAATTCACATGTGCGCAAATG
SV196F	GCTGCATTTAGGCTGCTACT
SV196R	CACCACTACCATGTCATGCT
SV359F	GGAGGTGCTATGCATTACCT
SV359R	TCAGCAAGCAGCCATTGTGT
SV403F	GGATGGTCCATGTCAGTCGT
SV403R	CGTCCTATCAGCACGTTCGT
SV406F	GGACAACATGAAGAAGTGGT
SV406R	GAGACTCTCCCACTTCGACT
SV407F	CACAGGAGTCCCAGAAGCCT
SV407R	CATCGTGAACGTCGGCAACT
SV409F	AGAGGAAGATGAAGACGCAC
SV409R	TGAGGATCTCTTCTGACTTCT

Table S2. *Cont.*

Marker	Primer Sequences (5′–3′)
SV410F	GTTGACTCACACGCACCATT
SV410R	GAATCTTAGAGGATGGAGGAC
SV411F	AGATCACCGCAAGCTTACATT
SV411R	AGATGGTAGTGATCAGCTGTT
SV412F	TGGAGCTCATCCATCAGGAT
SV412R	GTATGTCCATTGAGAGCGAG
SV413F	TGGGTCAGTCATCCAGTCAT
SV413R	CCTTGTTACCAGCACATTGC
SV415F	CTTGGAAGGCTCAATGTGGT
SV415R	AGCGAGTACCAGCTATAGGT
SV416F	CATGCCATCATCCTGCTGAT
SV416R	GGATCGGTTGAATATGCACGT
SV417F	ATAACACCTGCAGAGGGCGT
SV417R	GGTAGAGCATATCAGCAAGCT
SV418F	ACTAGGTTATTGCCCCCAAT
SV418R	GTAGCCACCTCACACATAGT
SV419F	AGCTCTGGACAGCAGCTGAT
SV419R	CCTCCCTTTCCATTGTGAACT
SV420F	CTATCCAATCTTGGAGTGCT
SV420R	CAAAGCCCAGAAGCACTCTC
SV421F	TTAGTCCAAGTTGCAGTTGTG
SV421R	CAAGAGATTTGACAAGGCTG
SV422F	TCGTGAGTGCTGATTCGAGT
SV422R	GACACTGCTGGAATACGTGT
SV426F	GCGAGTGCAGACCTATAGAT
SV426R	AAGGACTCAACAGTTCCACG
SV430F	CAAGCATGCAAGCCATATGGT
SV430R	TGCTCAGATGACATTGAGATG
SV501F	GTGGATCTCATGGAGCAGAC
SV501R	GGATGACGGAGAGGTAGAAC
SV503F	GGACAATCTCAGCTATGGTG
SV503R	TGTTTGCTCTGGAGGAGGAC
SV505F	GATCATCCACTCAGATCTGG
SV505R	ATGTAGGTACGACAACTTGTC
SV510F	GTGTTGTGAGACCCACAATG
SV510R	CAGCCATGGCCTACTTTCCT
SV518F	CTGTTCCATGAATTGCAATTCC
SV518R	GAAGCTGCCTGGTATCCAAT
SV521F	AGCCACACAGAGGTAAGAGT
SV521R	GGAGGCTGATTGACCCAAGT
SV563F	CCATGATAGGTGTGCAGTTG
SV563R	CAGGGTAAGTTCTCTTCACAC
SV602F	GTGTTAGGTGCACCAATGGT
SV602R	CTGTCTACTCTGACTGGACT

Table S2. *Cont.*

Marker	Primer Sequences (5′–3′)
SV701F	CAGCTGGTCAAGGATCTCAT
SV701R	GGTGTGAACTAATGTCCTAGG
SV702F	GCTCGAGCAAGACACAACAT
SV702R	TGTGGCTGTTAGGAGAGACT
SV703F	CCTGATTCATCTAGAGGTTGG
SV703R	ATCAGATGGTGTCTGGAGCT
SV706F	GAGGTAGCTTCCTTCATCAT
SV706R	CAGCAAGCAATCTACCAGGT
SV708F	CACTCACGGATCTGTCTTAC
SV708R	GTTGCTCTACCATCCTTAGT
SV901F	GTTTCCTGCCTGCAGGCAAG
SV901R	GACAGCTTGAAGGAGTTGAG
SV906F	GTCCATGGTATGCAGAGAAG
SV906R	CGAAGAGGAGGAGATGGAAT
SV907F	AATCTTCCAGCCAGCCAATG
SV907R	GAATGCGACCAAGGATCTGT
SV909F	AGCTCTGGACAGCAGCTGAT
SV909R	TCCATTGTGAACTACACAGAC
SV950F	CAGATTCTATCTCGGACTCC
SV950R	GATGAGGCAATCTGATGAGCT
SV952F	GTAGTGCTATGCTGAGCTGT
SV952R	GGAATGATGGTTGGCACTCT
SV953F	TTCCGAGTGCATGCCAGGAT
SV953R	GGAGTTGAGCAATCTCAGTC
SV954F	ATGCCACACCATGAGCACGT
SV954R	GTTCAACATGGCTAGCCATC
SV955F	TGGTCGTGGAACGAGAGTCT
SV955R	ACCTGATCAATGTGCTCGTC
SV957F	ACAGTTGTCGTGCTTCCTCT
SV957R	GGAAGGACAGGAACACGGAT
SV958F	GTGAGTAGGTCCATTCTCCT
SV958R	TGTGAGTGGCACTGCATGAT
SV959F	CACTGCTAGCCTAGACCGAT
SV959R	TCGTCGTAGTCAACATCGTC
SV960F	GGAGAATCAGCACCTCCAAG
SV960R	GGTCCTCAAACACTACACGT
SV961F	TGTGGTCCTACTGATGCGCT
SV961R	CTTCAAACTGATCTCCACTCT
SV962F	GCAATCACCTATCCTCTGG
SV962R	TTCTACCAGGTGTACCAGAT
SV963F	GATGACGTCACAGATATCGCT
SV963R	CGAAGGTGATCCTCCTTCCT
SV964F	TACCACCGTGACCAAGTCTT
SV964R	GTATGCCTGAGAGTCGAGAG

Table S2. *Cont.*

Marker	Primer Sequences (5′–3′)
SV966F	ATCAGACCATGCTCGTCCAC
SV966R	AGCTAACAGTCCTGTATTCTG
SV967F	AGCTGAGTGTGTACCTCTAT
SV967R	ACTGTTCACCACGGTATCAG
SV968F	TCCAAGACCATACGTCGAGT
SV968R	CCTGAGGTAGAATTGCATCC
SV969F	CGATACTCCTCACGTTTGAC
SV969R	ATCAGGGCATTGTGGAGAGT
SV971F	GTCACGAGTTGCTGAATGAG
SV971R	GTATGGAGTGATAGAGACCAC
SV972F	CACTCAAGACCTGTAGTCTC
SV972R	TTGACCGTCTTGATGCGAAG
SV973F	TTCCTCGTCCAGCAGAATCT
SV973R	CCTTGTCGATTCGTGGTTGT
SV974F	GGTGAGACTTGAAAGATTCG
SV974R	GGCAAGGATTATGCACCATTG
SV976F	TGCAGAGTGAGTGAGACGAT
SV976R	ACTAGGTGCTTGACGTAGAG
SV977F	ATTGATGCTGCACCAGTACC
SV977R	CAGAATGACTTGAGGCGTCT
SV978F	TGCGTAGGCATATGTTGCATT
SV978R	CAGGATGTTCCCATCACCAC
SV979F	GGACCGTAATGTAGGATAGC
SV979R	TGCGTATTGTCGATCACGTC
SV980F	TGCTCGTATGTGAACGTGAC
SV980R	GATCGAACTGAACCACAAGTC
SV981F	TCAGCATGTGTCAAGTATACAT
SV981R	TGTTGGTATAATCTTGGTTGTC
SV983F	TGAGCGAGATCACGTCCAGT
SV983R	CCAGAACGTTATCGACTGCT
SV984F	GAGGACATCGCATCATCCAC
SV984R	CCAGCCTATGCTTGTAGATG
SV985F	ACTCTCAAAGAGCATGACTC
SV985R	CACACTCGATGCATTAGGTG
SV987F	GCAGATGTGCATCAGCCATT
SV987R	CGTTGATGACAGACCAGATC
SV990F	CCAGTCACTTCTGAGACTCT
SV990R	CTCAGTTGTCGTTTGCGTCT
SV991F	GCAGCCTAGAGGAACTTTCT
SV991R	AGTCGTCATTGCTACTCCAG

Table S4. The primer sequences of three SCAR markers.

Marker	Primer Sequences (5′–3′)
SCAR 15F	TGGAGGGTGTTCCTATGGGTAAA
SCAR 15R	GGTGTTGGCGAACAAATTCTTAG
SCAR 48F	GTGTGCCCCAAGTGGAAGAA
SCAR 48R	GTGTGCCCCATACTCCCATATGA
SCAR 1270F	GGCGTATGGTGATGAAGTCAACT
SCAR 1270R	GGCGTATGGTCCTATTTTAAGTT
